# A Further Investigation of Combined Mismatch Repair and BRAFV600E Mutation Specific Immunohistochemistry as a Predictor of Overall Survival in Colorectal Carcinoma

**DOI:** 10.1371/journal.pone.0106105

**Published:** 2014-08-25

**Authors:** Nathan Luey, Christopher W. Toon, Loretta Sioson, Adele Clarkson, Nicole Watson, Carmen Cussigh, Andrew Kedziora, Stuart Pincott, Stephen Pillinger, Justin Evans, John Percy, Alexander Engel, Margaret Schnitzler, Anthony J. Gill

**Affiliations:** 1 Sydney Medical School, University of Sydney, Sydney, NSW, Australia; 2 Cancer Diagnosis and Pathology Research Group, Kolling Institute of Medical Research, St Leonards, NSW, Australia; 3 Histopath Pathology, North Ryde, NSW, Australia; 4 Sydney Vital Translational Research Centre, Royal North Shore Hospital, Pacific Highway, St Leonards, NSW, Australia; 5 Department of Anatomical Pathology, Royal North Shore Hospital, St Leonards, NSW, Australia; 6 Department of Colorectal Surgery, Royal North Shore Hospital, St Leonards, NSW, Australia; University of North Carolina School of Medicine, United States of America

## Abstract

Mutation specific immunohistochemistry (IHC) is a promising new technique to detect the presence of the BRAFV600E mutation in colorectal carcinoma (CRC). When performed in conjunction with mismatch repair (MMR) IHC, BRAFV600E IHC can help to further triage genetic testing for Lynch Syndrome. In a cohort of 1426 patients undergoing surgery from 2004 to 2009 we recently demonstrated that the combination of MMR and BRAFV600E IHC holds promise as a prognostic marker in CRC, particularly because of its ability to identify the poor prognosis MMR proficient (MMRp) BRAFV600E mutant subgroup. We attempted to validate combined MMR and BRAFV600E IHC as a prognostic indicator in a separate cohort comprising consecutive CRC patients undergoing surgery from 1998 to 2003. IHC was performed on a tissue microarray containing tissue from 1109 patients with CRC. The 5 year survivals stratified by staining patterns were: MMRd/BRAFwt 64%, MMRd/BRAFV600E 64%, MMRp/BRAFwt 60% and MMRp/BRAFV600E 53%. Using the poor prognosis MMRp/BRAFV600E phenotype as baseline, univariate Cox regression modelling demonstrated the following hazard ratios for death: MMRd/BRAFwt HR = 0.71 (95%CI = 0.40–1.27), p = 0.31; MMRd/BRAFV600E HR = 0.74 (95%CI = 0.51–1.07), p = 0.11 and MMRp/BRAFwt HR = 0.79 (95%CI = 0.60–1.04), p = 0.09. Although the findings did not reach statistical significance, this study supports the potential role of combined MMR and BRAF IHC as prognostic markers in CRC.

## Introduction

The development of biomarkers to predict outcome after definitive treatment of malignancy is an area of active research. Despite literally thousands of biomarkers having been explored in various cohorts, [1] very few have entered routine clinical practice. Reasons for the failure to translate into clinical care include cost, impracticality, lack of availability and failure of validation in different cohorts or across diverse populations. [1] An ideal biomarker would be inexpensive, readily deployable in the routine clinical setting and add genuine prognostic information in addition to that already provided by simple measures such as age, stage and grade.

In many institutions patients with colorectal carcinoma (CRC) undergoing surgery with curative intent are routinely offered reflex immunohistochemistry (IHC) for the DNA mismatch repair (MMR) proteins MLH1, PMS2, MSH2 and MSH6 in order to triage formal molecular testing for Lynch Syndrome. [2] We recently demonstrated, in a single institution cohort of patients undergoing surgery for CRC at Royal North Shore Hospital between calendar years 2004 and 2009, that the addition of BRAFV600E mutation specific IHC to MMR IHC holds promise as a biomarker for all cause survival. [3] This approach identifies the poor prognostic group of mismatch repair proficient (MMRp) BRAFV600E mutant CRC which accounted for 6.4% of CRC in our previous study. [3] Because the presence of BRAFV600E determination by either molecular means or IHC virtually excludes Lynch Syndrome in mismatch repair deficient (MMRd) CRC and is therefore commonly performed in many institutions in MMRd CRC, [2] this approach requires minimal extra expense and fits well into routine laboratory workflow.

In this study we sought to validate the combination of MMR and BRAFV600E IHC as a prognostic marker in CRC by examining its prognostic power in a different cohort – namely all patients undergoing surgery for CRC at the same institution from June 1998 to 2003.

## Materials and Methods

### Patients

We searched the database of the Department of Anatomical Pathology, Royal North Shore Hospital, for all patients who underwent surgery for CRC with curative intent from June 1998 to the end of calendar year 2003. During this period this department provided a centralized pathology service for 2 major quaternary centres with dedicated colorectal surgery units as well as four community hospitals with general surgery units. Patients treated endoluminally, with histologies other than adenocarcinoma or with tissue blocks unavailable for review were excluded. The pathology reports of all cases were reviewed (and if necessary the slides from cases were retrieved and reassessed) in order to stage the tumours according to the AJCC 7^th^ edition 2009 staging system [4].

### Immunohistochemistry

Tissue microarrays (TMAs) containing two 1 mm cores of carcinoma were created. IHC for the MMR associated proteins MLH1, PMS2, MSH2 and MSH6 was performed and interpreted using standard and previously described methods. [6] BRAFV600E mutation specific IHC was performed using a commercially available mouse monoclonal antibody (clone VE1, SpringBioscience, Pleasonton CA) using the same methods we have previously described. [2], [3], [5] Briefly VE1 IHC was performed using the Leica BondIII autostainer (Leica Microsystems, Mount Waverley, VIC, Australia) used according to the manufacturer’s protocol with alkaline antigen retrieval (solution ER2, VBS part no: AR9640, Leica Microsystems) with the primary antibody being used at a dilution of 1 in 80. BRAFV600E staining was interpreted as positive if >20% of neoplastic cells stained positively. The presence of definitive negative staining for any one of the four MMR markers was interpreted as evidence of mismatch repair deficiency (MMRd). MMR and BRAFV600E IHC was interpreted by observers who were blinded to all other clinical and pathological data.

### Survival Data

Follow up data was obtained by examination of hospital medical records and the hospital pathology database, assessment of records from surgeons’ private rooms and examination from publicly available death notices up to January 2014. Overall survival was defined as the duration alive from time of definitive surgery. In patients with metachronous CRCs, survival was taken from the time of surgery for the first CRC with subsequent tumors (either recurrences or second primary tumors) being excluded from survival analysis.

### Statistical analysis

Single variable p-values were computed using either the chi-square test for categorical variables or the Mann-Whitney-U test for scalar variables such as age at diagnosis. Five year survival values were obtained via Kaplan Meier analysis for each of the four MMR/BRAF IHC tumour phenotypes.

The effect of MMR/BRAF tumour IHC phenotype on overall survival was explored using Cox regression proportional hazards analysis, including a final model adjusted for gender, age at diagnosis, anatomic location, histologic grade and overall stage.

A p-value of <0.05 was taken as significant. All analysis was performed using IBM SPSS statistics for MAC, Version 21.0 (IBM Corp, Armonk NY USA, released 2012).

This study was approved by the Northern Sydney Local Health District Human Research Ethics Committee under protocol 1201-035 M. The ethics committee waived the need for consent to use the archived formalin fixed paraffin embedded tissue blocks and to access medical records on the basis that the study was only performed on archived formalin fixed paraffin embedded tissue removed during routine care many years previously. All patient information was anonymized and de-identified prior to analysis.

## Results

A total of 1109 colorectal carcinomas met inclusion criteria and had cores available in the TMA sections. The clinical and pathological details are presented in Table 1. Briefly, the median age at diagnosis was 72 years, 49.2% were female, and 75% had stage 2 or 3 disease. 856 patients were MMRp (85.9%) of which 133 (13.4% of the total) were MMRp/BRAFV600E mutant and 720 (72.5% of the total) were MMRp/BRAFwt. 144 were MMRd (14.1%), of which 108 (10.9% of the total) were MMRd/BRAFV600E and 32 (3.2% of the total) were MMR-deficient/BRAF wild type.

**Table 1:** Clinical and pathological features of 1109 consecutive patients with CRC.

Variable	Count (%)	SingleVariablep-value	Univariateanalysis HR(95%CI),p-value	Multivariateanalysis HR(95%CI),p-value
**Gender**		0.63		
female	546 (49.2)		1.00	1.00
male	563 (50.8)		1.15 (0.96–1.37), 0.13	1.22 (1.00–1.50), 0.05
**Age at diagnosis**	72 (28–100)	N/A	1.05 (1.04–1.05), <0.01	1.05 (1.04–1.06), <0.01
**Anatomic location**		<0.01		
rectum	278 (25.5)		1.00	1.00
caecum	156 (14.3)		1.17 (0.86–1.59), 0.32	0.85 (0.60–1.21), 0.37
ascending colon	218 (20.0)		1.16 (0.89–1.50), 0.28	0.87 (0.64–1.18), 0.37
transverse colon	130 (11.9)		1.23 (0.91–1.67), 0.18	0.80 (0.55–1.15), 0.23
descending colon	48 (4.4)		0.99 (0.63–1.55), 0.97	0.90 (0.54–1.48), 0.67
sigmoid colon	260 (23.9)		1.19 (0.93–1.54), 0.17	1.04 (0.79–1.38), 0.78
**Histologic grade**		<0.01		
low	835 (80.9)		1.00	1.00
high	197 (19.1)		1.39 (1.11–1.75), <0.01	1.13 (0.87–1.47), 0.36
**AJCC Stage**		<0.01		
I	207 (18.7)		1.00	1.00
IIA	295 (26.6)		0.28 (0.17–0.46), <0.01	0.10 (0.05–0.20), <0.01
IIB	54 (4.9)		0.35 (0.21–0.57), <0.01	0.13 (0.06–0.25), <0.01
IIC	15 (1.4)		0.95 (0.53–1.69), 0.86	0.36 (0.17–0.79), 0.01
IIIA	40 (3.6)		1.06 (0.48–2.33), 0.90	0.32 (0.12–0.81), 0.02
IIIB	321 (28.9)		0.31 (0.15–0.61), <0.01	0.15 (0.06–0.34), <0.01
IIIC	107 (9.6)		0.63 (0.39–1.01), 0.06	0.22 (0.11–0.44), <0.01
IVA	39 (3.5)		1.49 (0.89–2.50), 0.13	0.59 (0.28–1.22), 0.16
IVB	5 (0.5)		2.23 (1.26–3.98), <0.01	1.30 (0.60–2.83), 0.50
**MMR IHC status**		<0.01		N/A
proficient (MMRp)	856 (85.9)		1.00	
deficient (MMRd)	140 (14.1)		0.90 (0.69–1.17), 0.42	
**BRAF IHC status**		<0.01		N/A
wild type (BRAFwt)	774 (76.2)		1.00	
mutant (BRAFV600E)	242 (23.8)		1.12 (0.91–1.38), 0.30	
**MMR/BRAF IHC phenotype**		<0.01		
MMRp/BRAFV600E	133 (13.4)		1.00	1.00
MMRd/BRAFwt	32 (3.2)		0.71 (0.40–1.27), 0.25	1.12 (0.61–2.06), 0.72
MMRd/BRAFV600E	108 (10.90		0.74 (0.51–1.07), 0.11	0.87 (0.58–1.31), 0.51
MMRp/BRAFwt	720 (72.5)		0.79 (0.60–1.04), 0.09	0.80 (0.60–1.08), 0.15

Table 2 presents the overall survival figures for each of the four MMR/BRAF phenotypes as determined by the Kaplan Meier analysis. The 5-year survivals were 52.6% for MMRp/BRAFV600E, 64.2% for MMRd/BRAFwt, 64.1% for MMRd/BRAFV600E and 60.1% for MMRp/BRAFwt CRCs.

**Table 2:** Overall survivals of each of the four MMR/BRAF phenotypes by Kaplan Meier actuarial analysis.

MMR/BRAF phenotype	5-year survival	Mean survival
MMRp/BRAFV600E	52.6%	7.12 years (95%CI = 5.87–8.37)
MMRd/BRAFwt	64.2%	8.36 years (95%CI = 6.16–10.56)
MMRd/BRAFV600E	64.1%	8.08 years (96%CI = 7.04–9.40)
MMRp/BRAFwt	60.1%	8.06 years (95%CI = 7.62–8.50)

**Figure 1:**
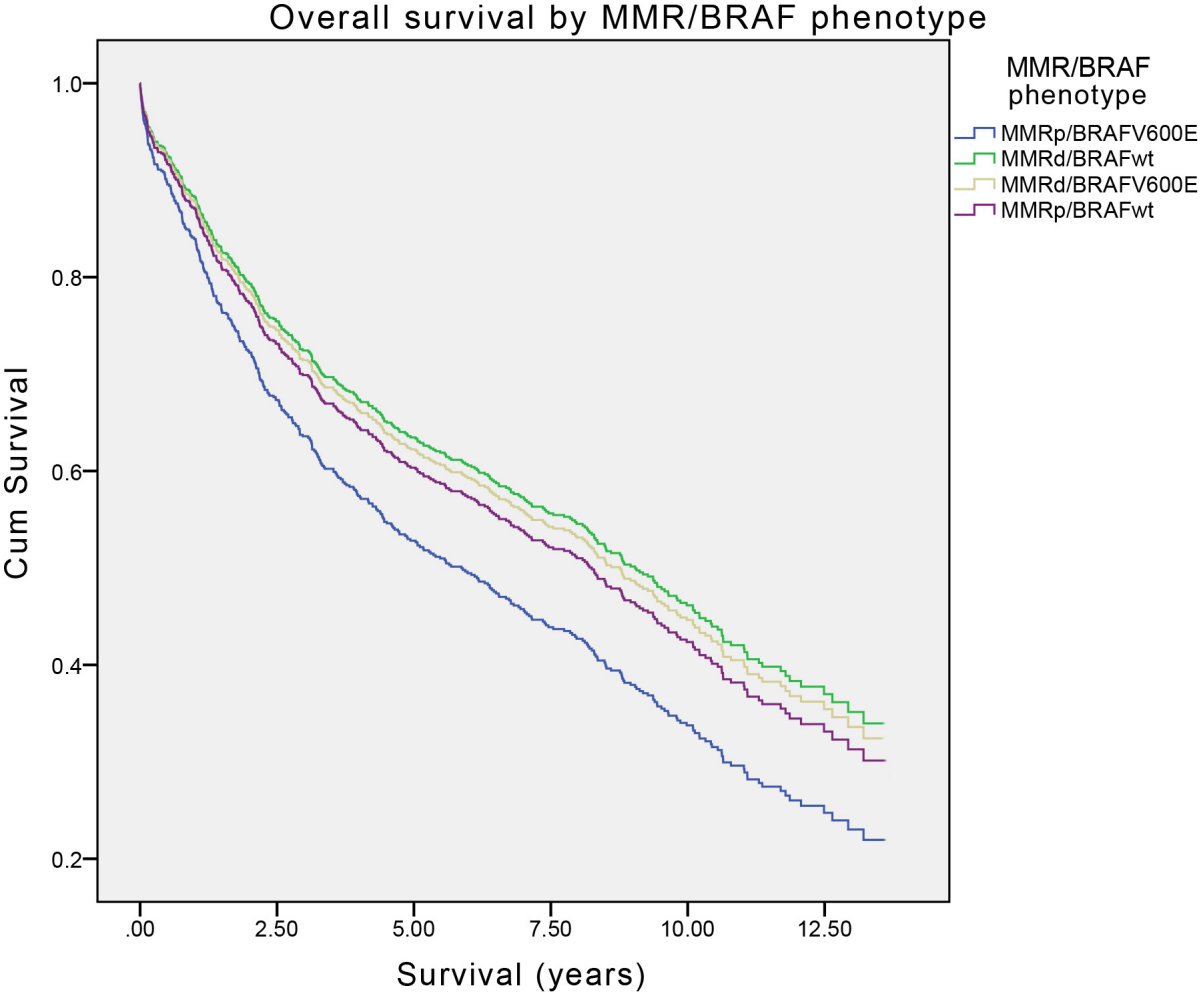
Overall survival of patients with CRC stratified by MMR and BRAF status (Cox regression modelling).

The univariate Cox regression survival function demonstrating the crude (unadjusted) relationship between survival and MMR/BRAF status is presented in Table 1 and Figure 1. Tumours segregated according to their MMR/BRAF phenotype in a consistent trend throughout the period of follow-up, with the MMRp/BRAFV600E trending towards worse prognosis compared to the other three phenotypes. Compared to the MMRp/BRAFV600E phenotype, tumours displaying the MMRp/BRAFwt phenotype tended towards significantly improved survival with a hazard ratio of 0.79 (95%CI = 0.60–1.04, p = 0.09). This effect was markedly diminished in the multivariate model due to the dominant effects of gender, age at diagnosis and tumour stage on overall survival (adjusted effect).

## Discussion

The determination of BRAF mutation status by immunohistochemistry has the significant advantages over molecular techniques of being both inexpensive and fitting easily into standard surgical pathology workflow. In laboratories where all CRCs routinely undergo screening for Lynch Syndrome with MMR IHC, the addition of BRAFV600E mutation specific immunohistochemistry would simply be a matter of performing IHC for 5 rather than 4 markers entailing minimal extra labour or handling costs and would have the added advantage of further triaging molecular testing for Lynch Syndrome in MMRd CRC. Therefore, if the addition of BRAFV600E mutation specific IHC to all CRCs can be validated as a biomarker, there is real potential that it may become the first prognostic biomarker for CRC deployed into routine clinical practice.

When we previously investigated the prognostic power of combined MMR and BRAFV600E IHC in a group of 1426 CRC from 2004 to 2009 [3], univariate analysis demonstrated that MMRp/BRAFV600E CRCs had a statistically significantly worse outcome compared to the other phenotypes (hazard ratio of 1.79 (95%CI = 1.24–2.60), (p)<0.01). This result was negated in multivariate analysis (hazard ratio of 1.10 (95%CI = 0.69–1.76), (p) = 0.68) primarily due to the dominant effect of stage and age on overall survival. In the current study, comprising CRCs from 1109 from the same institution resected from 1998 to 2003, MMRp/BRAFV600E CRCs trended towards a worse prognosis compared to all other tumour groups but failed to gain statistical significance (MMRd/BRAFwt p = 0.31, MMRd/BRAFV600E p = 0.11 and MMRp/BRAFwt p = 0.09). Whilst our findings support the prognostic utility of the combination of MMR and BRAFV600E IHC, the failure to achieve statistical significance indicates that further studies in larger cohorts will be needed to validate this approach. Ideally such further studies should be in truly independent external cohorts (that is from other institutions) rather than merely representing a preceding cohort from the same institution as in this case.

To date 13 studies have directly compared the accuracy of BRAF mutation status determination by IHC with molecular techniques. In 11 studies BRAFV600E mutation specific IHC has either outperformed or performed comparably to molecular techniques [2], [7], [8], [9], [10], [11], [12], [13], [14],[15],[16] whereas in two studies mutation specific IHC was found to be less reliable. [17], [18] A fair reading of the literature would support the approach taken by Kuan et al, [12] that mutation specific IHC is reliable but requires rigorous technical optimization and ongoing quality assurance including the performance of molecular testing in equivocal cases. Whilst this study was not intended or designed to assess the accuracy of BRAFV600E mutation specific IHC we note that the antibody has previously been proven to be extremely reliable in our hands. [2] The overall rate of BRAF mutation as determined by IHC in this study (23.8%) is in keeping with the 18.4% incidence we reported in a similar cohort of consecutiveCRCs from 2011 from the same institution tested by molecular means alone [2].

Although there are limitations to this study, most importantly that it did not represent a true external validation cohort but rather a validation cohort from the same institution, our finding of a trend towards survival differences amongst CRC when stratified by BRAFV600E and MMR IHC status is very similar to that which we have previously reported. [3] This provides cautious support to the use of a combination of BRAFV600E and MMR IHC as prognostic biomarkers in CRC. Ultimately similar studies will need to be performed in large truly independent cohorts before this approach can be considered validated. In the interim, the combination of BRAFV600E and MMR IHC still has a clear role in the triaging of patients with CRC encountered in routine clinical practice for formal molecular testing for Lynch Syndrome. [2], [7], [8], [9], [10], [11], [12], [13], [14], [15], [16] The strong likelihood that this approach can also have the added benefit of predicting outcome can be considered a likely downstream benefit of universal screening for Lynch Syndrome by immunohistochemistry.
